# Supplementary material for: Transgelin, a p53 and PTEN-Upregulated Gene, Inhibits the Cell Proliferation and Invasion of Human Bladder Carcinoma Cells In Vitro and In Vivo
Source: Int J Mol Sci. 2019 Oct 7;20(19):4946. doi: 10.3390/ijms20194946 (PMC6801752; doi:10.3390/ijms20194946)
Supplement: Supplementary file 1 [file ijms-20-04946-s001.pdf]

Supplementary Figures

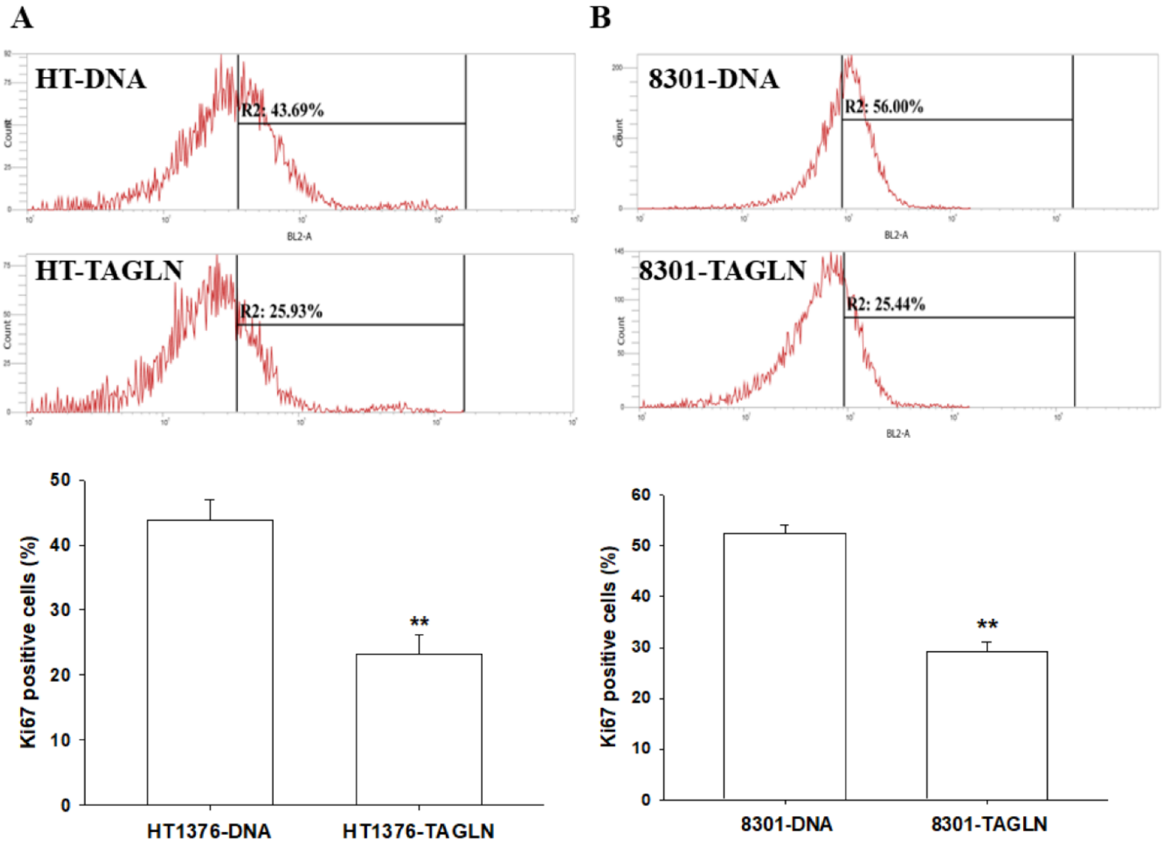

Figure S1. Effect of TAGLN on cell proliferation of bladder carcinoma HT1376 and TSGH-8301 cells determined by Ki-67 assays. Proliferation rates between (**A**) mock-transfected HT1376 (HT-DNA) and ectopic TAGLN overexpressed HT1376 (HT-TAGLN), and (**B**) between mock-transfected TSGH-8301 (8301-DNA) and ectopic TAGLN overexpressed TSGH-8301 (8301-TAGLN) cells were determined by Ki-67 assays (± SE; $n = 3$). ** is represented the $p < 0.01$.

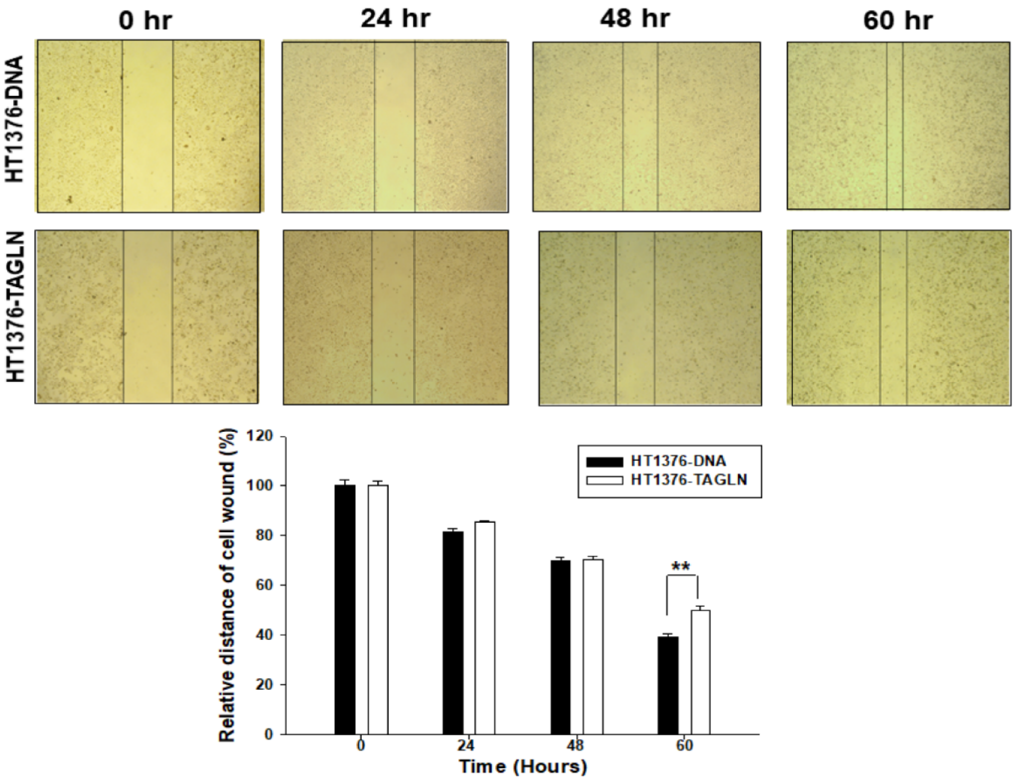

Figure S2. Effect of TAGLN on cell migration of bladder carcinoma HT1376 cells. The migration ability of HT-DNA and HT-TAGLN cells was determined by wound healing assays during the indicated times. ** is represented the $p < 0.01$.
